# Strong associations between plant genotypes and bacterial communities in a natural salt marsh

**DOI:** 10.1002/ece3.4105

**Published:** 2018-04-24

**Authors:** Gregory P. Zogg, Steven E. Travis, Daniel A. Brazeau

**Affiliations:** ^1^ Department of Biology University of New England Biddeford Maine; ^2^ Department of Biomedical Sciences University of New England Biddeford Maine

**Keywords:** bacteria, growth form, plant genotypes, plant–soil (below‐ground) interactions, rhizosphere, salt marsh, *Spartina alterniflora*

## Abstract

Although microbial communities have been shown to vary among plant genotypes in a number of experiments in terrestrial ecosystems, relatively little is known about this relationship under natural conditions and outside of select model systems. We reasoned that a salt marsh ecosystem, which is characterized by twice‐daily flooding by tides, would serve as a particularly conservative test of the strength of plant–microbial associations, given the high degree of abiotic regulation of microbial community assembly resulting from alternating periods of inundation and exposure. Within a salt marsh in the northeastern United States, we characterized genotypes of the foundational plant *Spartina alterniflora* using microsatellite markers, and bacterial metagenomes within marsh soil based on pyrosequencing. We found significant differences in bacterial community composition and diversity between bulk and rhizosphere soil, and that the structure of rhizosphere communities varied depending on the growth form of, and genetic variation within, the foundational plant *S. alterniflora*. Our results indicate that there are strong plant–microbial associations within a natural salt marsh, thereby contributing to a growing body of evidence for a relationship between plant genotypes and microbial communities from terrestrial ecosystems and suggest that principles of community genetics apply to this wetland type.

## 1 INTRODUCTION

Understanding plant–microbial interactions is essential to advancing our knowledge of the mechanisms by which biodiversity regulates ecosystem processes, as well as predicting responses to environmental change, due to the intimate relationship between producers and decomposers (Hooper et al., 2000). Plants have a particularly close association with microbes belowground because root exudates are important sources of carbon and other nutrients that heterotrophic microbes require (Hartmann, Schmid, Tuinen, & Berg, 2009). Given that plant species differ in the quantity and quality of their belowground inputs to soil (Wardle et al., 2004), the structure of their rhizosphere microbial communities can be distinctive, in terms of both composition and diversity (e.g., Costa et al., 2006; Garbeva, van Elsas, & van Veen, 2008). Furthermore, the influence of plants can extend beyond the rhizosphere due to above‐ and below‐ground litter production, as well as physical alterations of soil (Prescott & Grayston, 2013).

Genetic variation among individual plants also has the potential to influence microbial community structure (Schweitzer, Madritch, Felker‐Quinn, & Bailey, 2012), particularly in species depauperate ecosystems where genotypic diversity can serve as a surrogate for species diversity (Whitham et al., 2006). For example, in terrestrial ecosystems dominated by trees in the genus *Populus,* genotypes have been shown to vary in a number of phenotypic traits that could effect microbes, such as litter chemistry (Madritch, Greene, & Lindroth, 2009; Schweitzer et al., 2004) and productivity (Lojewski et al., 2009), and as a result, distinct microbial communities have been observed in bulk soil associated with different genotypes in a number of experiments (Madritch & Lindroth, 2011; Schweitzer et al., 2008). Similarly, variation in rhizosphere microbial communities among genotypes has been demonstrated experimentally for *Arabidopsis thaliana* (Lundberg et al., 2012; Micallef, Shiaris, & Colón‐Carmona, 2009) and has been attributed to differences among genotypes in root exudates (Micallef et al., 2009). However, relatively little is known about the relationship between plant genotypes and microbial communities outside of these model systems and under natural conditions (Schweitzer et al., 2011).

We reasoned that a salt marsh ecosystem, which is characterized by twice‐daily flooding by tides, would serve as a particularly conservative test of the strength of plant–microbial associations, given the potentially high degree of abiotic regulation of microbial community assembly resulting from alternating periods of inundation and exposure. Within a natural salt marsh in the northeastern United States, we characterized genotypes of the foundational plant *Spartina alterniflora* using microsatellite markers, and microbial metagenomes within marsh soil based on pyrosequencing. We first compared the composition and diversity of bacterial communities between bulk and rhizosphere soil, and then between rhizosphere soil associated with the two dominant growth forms of *S. alterniflora* (so‐called short form and tall form, Valiela, Teal, & Deuser, 1978), in order to determine if plants had any influence over microbes in this system. Most importantly, we explored whether rhizosphere bacterial communities were more similar beneath closely related plants and clonal ramets than beneath more genetically distinct plants. We predicted that there would be a strong association between plant genotypes and microbes in their rhizosphere because of previously documented morphological variation within *S. alterniflora* (e.g., Hester, Mendelssohn, & McKee, 1998; Hughes, 2014; Proffitt, Travis, & Edwards, 2003; Seliskar, Gallagher, Burdick, & Mutz, 2002). Insight into the factors that determine microbial community structure within these tidal wetlands is relevant to efforts to predict their response to anticipated rises in sea level, because microbial‐mediated decomposition contributes to the maintenance of marsh surface elevation (Charles & Dukes, 2009; Miller, Neubauer, & Anderson, 2001).

## 2 MATERIALS AND METHODS

Our study site was a macrotidal salt marsh, with organic matter‐rich soils (approximately 20% organic matter by weight), at the Wells National Estuarine Reserve in Wells, ME, USA (43.335295°N, 70.543490°W). We sampled at two locations during low tide in August 2010: one in an area containing tall *Spartina alterniflora* that was adjacent to a tidal creek (hereafter referred to as “tall form”); and one comprised of shorter *S. alterniflora* plants*,* located in the interior of the marsh and at a higher elevation (“short form”). Within both locations, we collected 12 individual *S. alterniflora* plants and their associated soil (10 cm diameter × 15 cm depth) in a 3 × 4 array (25 cm spacing between plants). The samples were transported on ice to the laboratory (transit time = 27 min), taking special care to insure that soil cores remained intact.

Approximately 0.1 g of tissue was collected from each plant and stored at −80°C for subsequent plant genotyping. Approximately 1 g of soil was scraped from the surface of a root from each individual plant (hereafter referred to as “rhizosphere” soil), and another 1 g was collected 5 cm away from the nearest rhizome (“bulk” soil). Both rhizosphere and bulk soil samples were collected using a sterile technique and stored at −80°C for subsequent microbial characterization. Although pyrosequencing was conducted on DNA extracts from all soil samples (the first step in the microbial characterization process), metagenomic analyses were conducted on only a select subset of these samples, chosen based upon the plant genotyping results (see Section 2.2 below for specifics).

### 2.1 Plant genotyping and microbial pyrosequencing

We determined the genotypic identity of plants on the basis of neutral microsatellite markers. We first extracted DNA from leaf tissue using Qiagen's DNeasy Plant Mini Kit (Germantown, MD, USA) according to the manufacturer's protocol. We PCR‐amplified four microsatellite loci developed from *S. alterniflora* DNA by Blum, Sloop, Ayres, and Strong (2004) according to the recommended protocols, including SPAR02, 03, 05, and 09, which were chosen for their high allelic diversity. Microsatellite alleles were resolved by electrophoresis on an ABI PRISM 310 Genetic Analyzer (Applied Biosystems, Inc., Foster City, CA, USA) and sized using Genemapper, Version 4.0. We determined genet identities by matching alleles among ramets within each location. Note that because of the hexaploid nature of *S. alterniflora*, and the extreme allelic diversity of the microsatellite loci used in identifying genets (up to 12 alleles per locus among the 24 ramets included in the study), the probability of ramets from distinct genets matching by chance was negligible.

We characterized microbial metagenomics within soil samples on the basis of pyrosequencing of 16S rDNA (after Kim et al., 2008). Soil samples were extracted using the Powersoil DNA isolation kit for soils from MO‐BIO (Carlsbad, CA, USA). Approximately 100 ng of DNA from each sample was PCR‐amplified using primers targeting regions flanking the variable regions 1 through 3 (V1–V3) of the bacterial 16S rRNA gene (table 1 in Wu et al., 2010). Each primer set used for PCR included one of six unique ten‐base pair barcodes to allow multiplexing of six samples in each sequencing run. PCR amplification consisted of 50‐μl reactions containing 3.0 mmol/L MgCl_2_, 0.1 μmol/L each primer, 200 μmol/L each dNTP, and 0.02 Units of iProof High‐Fidelity DNA Polymerase (BioRad, Hercules, CA, USA). The thermocycle consisted of an initial denaturation at 98°C for 30 s, followed by 35 cycles of 98°C for 10 s, 58°C for 30 s, and 72°C for 60 s, followed by a final extension at 72°C for 7 min. Amplicons were pooled into eight libraries (six samples per library) and purified using the Agencourt AMPure XP system (Beckman Coulter, Brea, CA, USA). Libraries were quantified using KAPA library Quantification for Roche 454 GS sequencing (KAPA Biosystems, Boston, MA, USA). Pyrosequencing was conducted using the 454/Roche GS Junior (Roche 454 Life Sciences, Branford, CT, USA) and carried out according to the manufacturer's instructions for amplicon libraries.

### 2.2 Metagenomic annotation and data analysis

Based upon our plant genotyping, we identified 12 distinct plant genets, or clones: seven for tall form *S. alterniflora* and five for short form. We selected four plants of each type that were distinct clones—as well as a fifth plant that was a ramet of one of the four clones of that growth form—and evaluated the microbial metagenomes within the rhizosphere and bulk soil associated with these plants. Taxonomic classification was conducted in MG‐RAST (Meyer et al., 2008) using the Greengenes annotation (DeSantis et al., 2006) and the following cutoff parameters: maximum *e*‐value of 1*e*−5, minimum identity of 60%, and minimum alignment length of 15 bp. As a further quality control check, any bulk or rhizosphere samples that fell below the 95% confidence interval for either total number of base pairs or sequences, across all metagenomes, were omitted (resulting sample sizes for each analysis are given in parentheses below). Metagenomes were dominated by organisms in the Domain Bacteria (92.3% relative abundance, based upon 104,933 sequences with an average length of 301 bp). Only a small proportion was of Eukaryotic origin (1.0%, including diatoms in the Phylum Bacillariophyta, and green algae in the Chlorophyta and Streptophyta), or could not be assigned at the level of Domain (6.7%). Although the majority of the bacteria could be identified at lower taxonomic levels, including species, using the Greengenes annotation (DeSantis et al., 2006), over a third of the annotation hits (38.9% of the overall total) could not be identified below the level of Domain—that is, we had a large number of hits for entities like “unclassified (derived from Bacteria)” or “unclassified (derived from Bacteriodetes).” Thus, for all comparisons between metagenomes at the species level (described below), we used Spearman's coefficient (rho), which is based on ranks. This nonmetric correlation coefficient has been shown to be good at both identifying subtle clusters of microbial communities and detecting differences in communities across environmental gradients, even when the number of sequences per sample is small (Kuczynski et al., 2010, and supplemental tables therein).

In order to determine the relationship between plants and microbial communities, we compared metagenomes between bulk and rhizosphere soil samples collected from the same plant (*n* = 12 soil samples total, one of each type from six different plants that had metagenomes that met our selection criteria). First, we applied a principal coordinates (PCO) analysis to taxonomic data at the level of species and generated two‐dimensional ordination plots to visualize the separation between communities from the two soil types. In order to determine if there was a statistically significant difference in metagenomes from bulk versus rhizosphere soil, we used a nonparametric multivariate analysis of variance (PERMANOVA). Next, we sought to identify which bacterial taxa contributed to differences in metagenomic communities between soil types from heatmap plots at the level of Phyla, using Ward's hierarchical clustering method (clustering threshold of 0.75). Lastly, we evaluated whether or not diversity varied between rhizosphere and bulk soil by comparing the number of species between soil types with a *t* test.

We further explored the association between plants and microbes by examining patterns within rhizosphere samples exclusively (*n* = 10 soil samples total, half from short form and half from tall form plants). Differences in metagenomic communities between short‐form and tall‐form *S. alterniflora* were compared visually in the ordination space derived from a PCO analysis, and statistically with a PERMANOVA. We used a heatmap plot of Bacterial Classes, restricted to the most dominant Phyla identified in the bulk versus rhizosphere analysis, to examine which taxa contributed most to differences in rhizosphere metagenomes between short‐form and tall‐form plants. Species diversity was compared via a *t* test. In order to determine if rhizosphere microbial communities were more similar among closely related plants, we first constructed a matrix of plant genetic relatedness among all possible pairwise combinations of plants (10 comparisons for each growth form) based upon the method‐of‐moments (MOM) estimator of Huang, Ritland, Guo, Shattuck, and Li (2014), which was developed for use with polyploids such as *S. alterniflora*. An analogous matrix of microbial similarity was developed for rhizosphere metagenomes at the species level, based on rho coefficients for soil from the same plants as in the plant genetic relatedness matrix. Then, we determined whether the plant and microbial matrices for each plant form were significantly correlated by Mantel tests. We also examined variation among plant clones using values from the microbial matrix and concluded that microbial communities were significantly more similar beneath two ramets of the same clone than ramets of different clones if the rho coefficient between their metagenomes was outside the 95% confidence interval of values for all possible comparisons.

Spearman's rho coefficients, PCO analyses, and PERMANOVA tests were all computed using PAST software (v 3.12; Hammer, Harper, & Ryan, 2001). Heatmaps were generated in STAMP (v 2.1.3; Parks, Tyson, Hugenholtz, & Beiko, 2014), and t tests were conducted in SYSTAT (v13, San Jose, CA, USA). Similarity matrices were calculated for plants in PolyRelatedness (v1.5; Huang et al., 2014) and for soil metagenomes in PAST; Mantel tests were performed in PASSaGE (v2; Rosenberg & Anderson, 2011). An alpha of 0.05 was used for all statistical tests, and 999 permutations were used for any procedure that required them.

## 3 RESULTS

**Figure 1:**
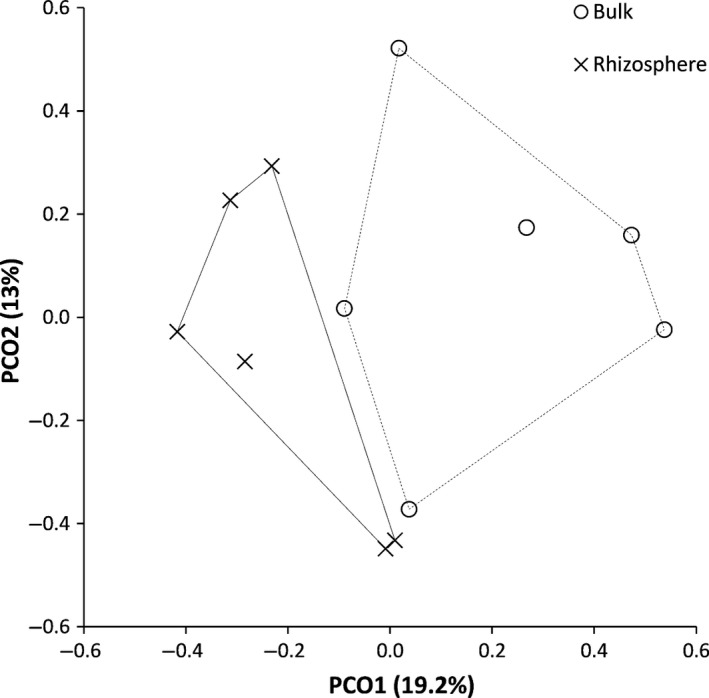
Differences in metagenomic communities between bulk and rhizosphere soil, based upon a Principle Coordinates Analysis (PCO) of taxonomic data at the level of species. Values in parentheses indicate percent of variation explained by axes PCO1 and PCO2, and lines are convex hulls around all points of a given soil type

**Figure 2:**
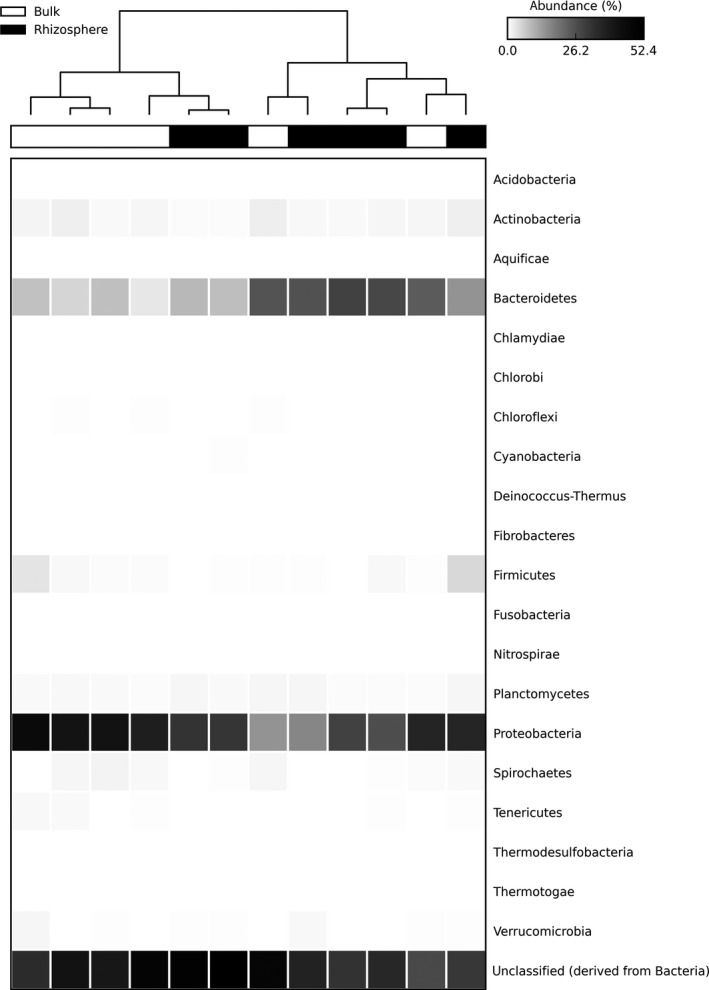
Heatmap comparison of sequence abundances for Phyla (Domain Bacteria) between metagenomes from bulk and rhizosphere soils

We found marked differences in metagenomes depending upon their proximity to the roots of *S. alterniflora*. Rhizosphere communities were clearly distinct from those in bulk soil in ordination space derived from a principle coordinates analysis (Figure 1) and were significantly different from one another (PERMANOVA: false‐F = 1.588, *p* = .020). A heatmap indicated that bacteria from two Phyla were dominant in both soil types (Figure 2), and that rhizosphere soil had a higher abundance of sequence reads for the Bacteriodetes (mean ± *SD* of 19 ± 8.8% for rhizosphere, 12 ± 8.9% in bulk soil) and lower percentages for Proteobacteria (29 ± 6.2% for rhizosphere, 36 ± 10.2% for bulk). Both soil types had similarly high values for unclassified reads derived from Bacteria (40 ± 8.4% for rhizosphere; 40 ± 7.5% for bulk). Species diversity was significantly higher (*t*‐test: *t* = 2.07, *df* = 10, *p* = .033) in rhizosphere soil (178 ± 34.0, mean ± *SD*) than in bulk soil (146 ± 17.5).

**Figure 3:**
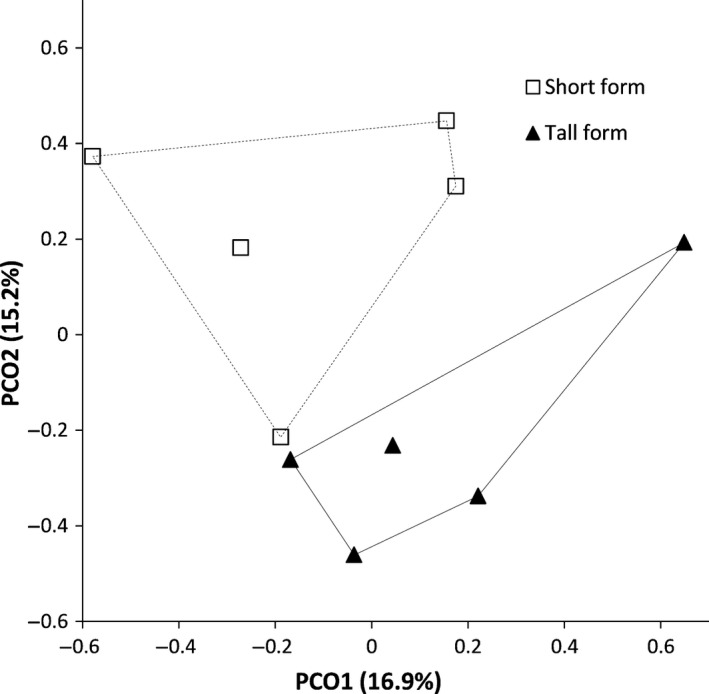
Differences in rhizosphere metagenomic communities between short and tall form *Spartina alterniflora* plants, based upon PCO analysis of taxonomic data at the level of species

**Figure 4:**
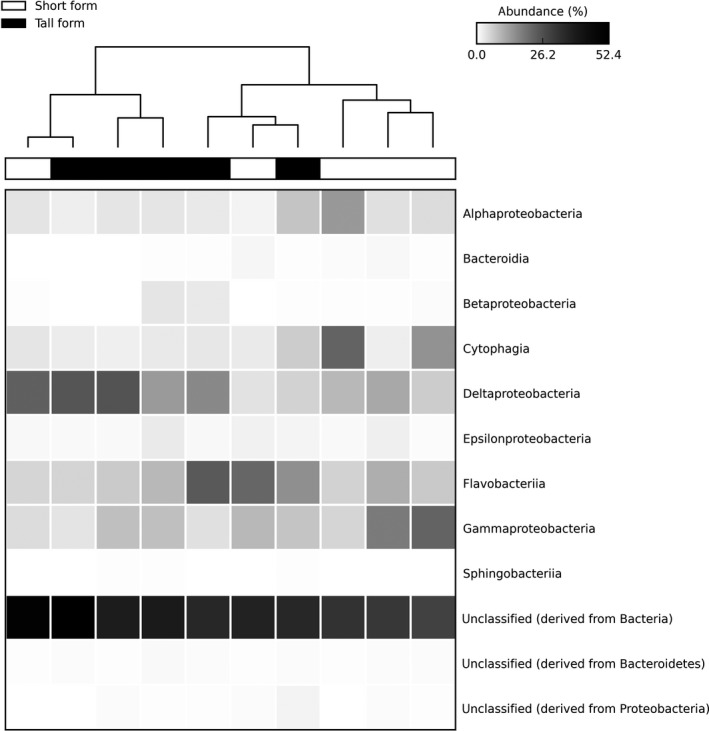
Heatmap comparison of sequence abundances for Classes between rhizosphere metagenomes from short‐form and tall‐form *Spartina alterniflora* plants. Shown only are data for the Phyla Bacteriodetes and Proteobacteria, as well as unclassified reads derived from Bacteria

Rhizosphere metagenomes associated with short‐form plants were distinct from those beneath tall form plants in two‐dimensional PCO ordination space (Figure 3) and were significantly different from one another (PERMANOVA: false‐F = 1.345, *p* = .016). A heatmap comparison, restricted to the dominant Phyla from the bulk versus rhizosphere comparison (Figure 4), indicated that rhizosphere bacteria were predominately from five Classes (with 5% or greater abundance in one of the growth forms). Metagenomes associated with tall form *S. alterniflora* had higher abundances of reads from Deltaproteobacteria (17 ± 7.5% for tall form, 11 ± 6.7% for short form) and Flavobacterilia (12 ± 6.9% for tall form, 10 ± 6.2% for short form); and lower abundances of Gammaproteobacteria (6 ± 2.2% for tall, 12 ± 7.2 for short), Cytophagia (3 ± 1.7% for tall, 9 ± 8.2 for short), and Alphaproteobacteria (4 ± 1.9% for tall form, 5 ± 4.2% for short form). Unclassified reads derived from Bacteria were also higher for tall form *S. alterniflora* (40 ± 6.3% for tall, 36 ± 7.6% for short). There was no significant difference in species diversity (*t* test: *t* = −0.73, *df* = 8, *p* = .487) between tall (210 ± 48.7) and short form plants (191 ± 32.2).

**Figure 5:**
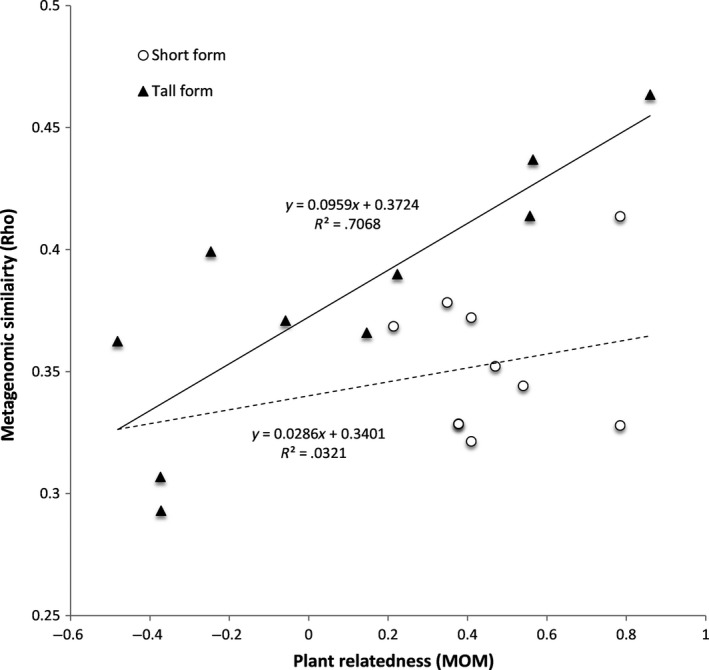
Correlations between rhizosphere metagenomic community similarity (Spearman's rho coefficient, Rho) and plant genetic relatedness (method‐of‐moment estimator, MOM). Plotted are all possible pairwise comparisons from short‐form and tall‐form *Spartina alterniflora* plants

Lastly, we found that rhizosphere metagenomes were more similar beneath closely related plants or clonal ramets than beneath more genetically distinct plants—but only for rhizospheres in association with tall form *S. alterniflora*. There was a significant, positive relationship between metagenomic similarity and plant relatedness for soil from tall form plants (Mantel test: *r* = .84, *p* = .043), but not for short‐form plants (Mantel test: *r* = .03, *p* = .951; see Figure 5 for a graphical representation). Furthermore, rhizome metagenomes beneath ramets from the same clone were significantly more similar to one another than beneath other plants, but only for rhizospheres associated with tall form plants (Rho = 0.463, which was outside the 95% C.I. of 0.343–0.367 of all possible pairwise comparisons), and not for short‐form plants (Rho = 0.344).

## 4 DISCUSSION

Although the assembly of marsh microbial communities is largely under abiotic control, our results indicate a strong association between plants and microbes within a natural salt marsh. Differences in microbial communities among marshes within a geographic region have been attributed to climatic variation (Blum, Roberts, Garland, & Mills, 2004; Martiny, Eisen, Penn, Allison, & Horner‐Devine, 2011). Spatial patterns within an individual marsh are regulated by hydrological conditions, as evidenced by studies that reported variation in community composition along gradients of elevation (Cao, Green, & Holden, 2008; Franklin, Blum, McComb, & Mills, 2002) and soil moisture content (Martiny et al., 2011). The potential importance of biotic factors has also been demonstrated, with marsh microbial communities having been shown to vary with percent cover of plants (Martiny et al., 2011) and among different plant species (Lovell & Davis, 2012). In our study, we found significant differences in both the composition and diversity of bacterial communities depending on their proximity to the roots of *S. alterniflora*. More importantly, we found that rhizosphere communities were more similar among genetically related plants or clonal ramets than beneath more genetically distinct plants.

Rhizosphere influences on microbial communities have been widely reported for terrestrial ecosystems and have been attributed primarily to carbon and other nutrients released by plant roots (reviewed by Hartmann et al., 2009). Within salt marshes, high levels of root oxygen production as a result of plant adaptations to anoxic conditions (e.g., aerenchyma and radial oxygen loss) can impact the microbial community as well (Bakker, Bouma, & van Wijnen, 2005). We suggest that both factors likely contributed to rhizosphere effects in our study. In particular, we observed significant differences in bacterial community composition between rhizosphere and bulk soil (Figure 1), as well as changes in the abundances of species in the two most common Phyla in our study: Bacteriodetes, which was higher in the rhizosphere; and Proteobacteria, which was higher in bulk soil (Figure 2). Elmer, Thiel, and Steven (2017) also found more Bacteriodetes in vegetated soil in a northeastern US marsh, which they attributed to an affinity for carbon production by *S. alterniflora*. Jiang et al. (2013) found higher abundances of Proteobacteria in the bulk soil of a tidal, saline, wetland dominated by mangroves, which they suggested was due to sulfur‐reducing bacteria adapted to the more anaerobic conditions outside the rhizosphere.

In contrast to most studies, where rhizosphere diversity is actually lower than in bulk soil (reviewed in Berendsen, Pieterse, & Bakker, 2012; de Vries & Wallenstein, 2017), presumably due to the ability of only a small number of microbes to successfully compete in rhizosphere conditions (Hartmann et al., 2009), we found significantly greater bacterial diversity in soil associated with plant roots. We suggest this difference may be due to the fact that the bulk soil environment in those studies, predominantly in terrestrial ecosystems, is relatively benign as compared to that in the harsh intertidal. *S. alterniflora* likely provides oxygen and labile organic compounds that a large number of microbes can utilize in an environment lacking in both, just as a desert plant functions as a “resource island” for microbes in a water and nutrient‐limited environment, and thus is predicted to have greater diversity within its rhizosphere (Herman, Provencio, Herrera‐Matos, & Torrez, 1995). Other studies of *S. alterniflora* found no rhizosphere effect on diversity (Hong et al., 2015), or even lower diversity in the rhizosphere (Zheng et al., 2016). Discrepancies between other studies and ours might be due to different methods—for example, Hong et al. (2015) used mudflat sediment for their nonrhizosphere sample, and Zheng et al. (2016) sampled only anammox communities—or because these other studies were conducted in Chinese wetlands where *S. alterniflora* is an invasive species. Future research in additional *S. alterniflora* marshes would be necessary to test if the “resource island” hypothesis applies more broadly.

Within the rhizosphere, we found significant differences in community composition depending on whether bacteria were collected from the roots of short‐form or tall‐form *S. alterniflora* (Figure 3). Other studies within northeastern US marshes have reported differences in bacteria among growth forms too, including the entire community in bulk soil (Bowen, Crump, Deegan, & Hobbie, 2009), and sulfur oxidizers in the rhizosphere (Thomas, Giblin, Cardon, & Sievert, 2014; although they did not explicitly mention growth forms, their Site 1 was located along the creekbank and thus presumably contained tall form plants, and their Site 2 was on the marsh platform so likely would have consisted of short‐form plants). The major taxonomic differences in our analysis were that rhizosphere microbes associated with tall form plants were dominated by bacteria in the Class Deltaproteobacteria, whereas short‐from microbes were predominantly from the Class Gammaproteobacteria (Figure 4). Microbes that are critical to sulfur cycling in marshes are found in both groups, with many sulfate‐reducing bacteria in the Deltaproteobacteria (Bahr et al., 2005), and the dominant sulfur oxidizers in the Gammaproteobacteria (Thomas et al., 2014). Differences in abiotic conditions between the locations where the two growth forms predominantly occur have been used to explain the greater stature of tall form plants—for example, due to higher nitrogen levels (Valiela & Teal, 1974) or better drainage (Mendelssohn, McKee, & Patrick, 1981) in tall form habitats—and thus could have directly impacted the structure of microbial communities associated with them in our study. For example, Thomas et al. (2014) attributed a greater abundance of Alphaproteobacteria sulfur oxidizers in the rhizosphere of tall form plants to lower sulfide availability for microbes, as a result of greater tidal flushing in tall form soil (their creekside Site 1).

We suggest that trait variation among growth forms might contribute to microbial patterns, in addition to abiotic factors, based upon evidence for genetic differences between short‐ and tall‐form plants from a common garden experiment (Gallagher, Somers, Grant, & Seliskar, 1988). In particular, short‐ and tall‐form *S. alterniflora* are known to differ in a number of root characteristics that could impact microbial communities, including higher iron content (Ornes, Sajwan, Loganathan, & Chetty, 1998) and a more oxidized rhizosphere (Mendelssohn & Postek, 1982) in tall form plants. The two growth forms also differ in the quantity and timing of belowground activity, with tall form plants producing large amounts of rhizomes and root exudates in the peak of the growing season (Jung & Burd, 2017), whereas short‐form plants have smaller and less episodic production (Hines, Knollmeyer, & Tugel, 1989). Consistent with this temporal pattern, and reports that maximum sulfate reduction rates occur earlier in tall form areas (Hines et al., 1989) than in locations dominated by short‐form plants (Howarth & Teal, 1979), we found greater abundances of Deltaproteobacteria in the rhizosphere of tall form plants at the period of peak biomass at our study site (i.e., August). Furthermore, Bowen et al. (2009) found that total bacterial composition did not differ between growth forms early in the growing season (May, June) when belowground activity would likely have been more similar, but was significantly different later in the year (July, August, September). However, we observed greater abundances of Gammaproteobacteria, which are adapted to relatively high oxygen levels (Thomas et al. (2014), within the short‐form rhizosphere, that cannot be readily explained by temporal differences in root activity between the growth forms nor by differences in abiotic conditions (i.e., we would expect lower numbers of these bacteria in short‐form habitat due to the more saturated soil, as compared to tall form locations).

As evidence for an association between the genetic structure of the plant population and the structure of the microbial community in marshes, we found that microbes in tall form *S. alterniflora* rhizospheres were more similar beneath ramets of the same clone, and among closely related plants, then beneath more genetically distinct plants. Our results contribute to a growing body of evidence for a relationship between plant genotypes and microbial communities from experiments in terrestrial ecosystems (e.g., Schweitzer et al., 2008 for *Populus spp*.; Lundberg et al., 2012 for *A. thaliana*). We are aware of only one such study conducted within salt marshes—a common garden experiment in China by Nie et al. (2010) which found that the composition and diversity of rhizosphere microbial communities varied depending upon the location of the source population of invasive *S. alterniflora*, and which they attributed to latitudinal variation in plant traits among populations. We suggest that our results, within a single marsh and under natural conditions, were due to trait variation within the native *S. alterniflora* population at our study site. We have previously observed within‐population genotypic variation in above‐ground characteristics among *S. alterniflora* clones collected from a single site (Proffitt et al., 2003), and variation in both above‐ and below‐ground traits have been reported among individuals collected from different native populations (Seliskar et al., 2002). Distinct genotypes in our study likely varied in below‐ground traits, such as root production and exudates, which caused microbial communities to be more similar among clonal ramets than other plants. The positive relationship between microbial community similarity and plant genetic relatedness that we observed for tall form plants (Figure 5) was likely due to greater similarity in trait characteristics among closely related individuals, such as has recently been demonstrated for root exudates from *A*. *thaliana* (Mönchgesang et al., 2016).

Interestingly, rhizosphere bacterial communities did not differ among short‐form genotypes, nor were they correlated with plant genetic relatedness in this location. Although we cannot definitively rule out abiotic factors as the cause of differences in the patterns between the two growth forms—for example, in the short‐form location, which has poorer drainage, less daily variation in anoxic conditions might have diminished the strength of plant–microbial relationships—we suggest that actual differences in the growth forms themselves, as described above, could have influenced these relationships as well. We have greater confidence that abiotic conditions, alone, cannot explain the correlation between microbial similarity and plant genetic relatedness within the tall form location, for two reasons. First, we purposely sampled in a very small area within each growth form location (0.375 m^2^), so as to minimize heterogeneity in the soil environment. Second, we indirectly examined the influence of abiotic factors by explicitly testing for a distance effect, with the assumption that abiotic conditions should be more similar the closer two sample locations are to one another. After constructing a matrix of spatial distances between all possible pairwise comparisons of plants (within a growth form), just as we did previously for plant relatedness, and comparing it to the microbial community similarity matrix, we found no significant relationship between sample distance and community composition for tall form plants (Mantel test: *r* = −.33, *p* = .161). However, our study design did not allow us to evaluate the relative importance of abiotic versus biotic factors in structuring microbial communities. We also only sampled in one short‐form and one‐tall form location within a single marsh, which presents the possibility that growth form differences we observed in microbial communities, or in the relationship between microbial similarity and plant genetic relatedness, were unique to our sample locations. Identifying the relative contribution of abiotic and biotic factors in structuring marsh microbial communities will require additional research, including field studies examining how microbial communities vary with both environmental conditions and root characteristics within short‐ and tall‐ form habitats, and experiments comparing microbes associated with short‐ and tall‐from plants grown in a common garden. Despite the foregoing caveats, our approach allowed us to identify some compelling in situ correlations between plants and microbes, which merit further investigation.

## 5 CONCLUSION

In summary, we found evidence for strong plant–microbial associations in a salt marsh, including differences in bacterial diversity and community composition between bulk and rhizosphere soil, and that the structure of rhizosphere communities varied depending on the growth form of, and genetic variation within, the foundational plant *S. alterniflora*. Although salt marshes have been identified as excellent systems for investigating the impact of plant genotypic diversity on other trophic levels (Reusch & Hughes, 2006), very little published work has examined this effect (e.g., on herbivore behavior aboveground by Zerebecki, Crutsinger, & Hughes, 2017; and on invertebrate abundance belowground by Hughes, 2014), and we aware of only one study, an experiment utilizing invasive *S. alterniflora,* that has looked at the response of microbes (Nie et al., 2010). Our results, from a field survey in a natural marsh, thus provide some novel and valuable insight into the strength of plant–microbial associations within this system and further suggest that principles of community genetics apply to this wetland type. Identifying the factors that determine microbial community structure is important because of growing awareness of how microbial structure influences ecosystem function and the response to environmental change (reviewed in Allison & Martiny, 2008; Bardgett & van der Putten, 2014). In the case of salt marshes, sea level rise poses a major threat to this ecosystem and the critical services that it provides (Craft et al., 2009). Given the contribution of microbial‐mediated decomposition to the maintenance of surface elevation in marshes (Kirwan & Blum, 2011), particularly in high latitude, organic matter‐rich sites like the one in the current study, microbial structure–function relationships have the potential to ultimately influence marsh survival (Simon, Zogg, & Travis, 2017). Thus more research into the relationship between plant genetic variation and microbial communities in marshes is warranted.

## CONFLICT OF INTEREST

None declared.

## AUTHOR'S CONTRIBUTIONS

GPZ and SET conceived of the project, designed the study, and collected the samples; SET conducted the plant genotyping and DAB performed the microbial pyrosequencing; GPZ led the writing of the manuscript. All authors contributed critically to data analysis and manuscript drafts.

## DATA ACCESSIBILITY

The microbial metagenomic data, labeled with plant number, clonal identity, growth form, and soil type, are publicly available on the MG‐RAST website (https://www.mg-rast.org/linkin.cgi?project=mgp84647).
